# Enhancing prebiotic, antioxidant, and nutritional qualities of noodles: A collaborative strategy with foxtail millet and green banana flour

**DOI:** 10.1371/journal.pone.0307909

**Published:** 2024-08-19

**Authors:** Tasnim Farzana, Md. Jaynal Abedin, Abu Tareq Mohammad Abdullah, Akter Hossain Reaz, Mohammad Nazrul Islam Bhuiyan, Sadia Afrin, Mohammed Abdus Satter

**Affiliations:** Bangladesh Council of Scientific and Industrial Research (BCSIR), Institute of Food Science and Technology (IFST), Dhaka, Bangladesh; University of Ha’il, SAUDI ARABIA

## Abstract

Foxtail millet **(**FM) and green banana (GB) are rich in health-promoting nutrients and bioactive substances, like antioxidants, dietary fibers, and various essential macro and micronutrients. Utilizing GB and FM flour as prebiotics is attributed to their ability to support gut health and offer multiple health benefits. The present study aimed to evaluate the impact of incorporating 10% GB flour (GBF) and different proportions (10–40%) of FM flour (FMF) on the prebiotic potential, antioxidant, nutrient, color, cooking quality, water activity and sensory attributes of noodles. The prebiotic potential, antioxidant, and nutrient of the produced noodles were significantly improved by increasing the levels of FMF. Sensorial evaluation revealed that noodles containing 30% FMF and 10% GBF attained comparable scores to the control sample. Furthermore, the formulated noodles exhibited significantly (*p* < 0.05) higher levels of protein, essential minerals (such as iron, magnesium, and manganese), dietary fiber (9.37 to 12.71 g/100 g), total phenolic compounds (17.81 to 36.35 mg GA eq./100 g), and total antioxidants (172.57 to 274.94 mg AA eq./100 g) compared to the control. The enriched noodles also demonstrated substantially (*p* < 0.05) increased antioxidant capacity, as evidenced by enhanced DPPH and FRAP activities, when compared to the control noodles. Overall, the incorporation of 30% FMF and 10% GBF led to a noteworthy improvement in the nutritional and antioxidant qualities of the noodles, as well as the prebiotic potential of the noodles with regard to *L*. *plantarum*, *L*. *rhamnosus*, and *L*. *acidophilus*. The implementation of this enrichment strategy has the potential to confer a multitude of health advantages.

## Introduction

The development of novel, nutritionally dense food products is approached with careful consideration for both physiological well-being and consumer feasibility. Noodles, being a culinary staple, hold a prominent position in the market due to their convenience in preparation, cost-effectiveness, extended shelf life, and widespread consumption [1–4]. Traditional noodle dough typically consists of wheat flour, water, and salt, but scientific analysis suggests that conventional noodle products may lack essential nutrients like dietary fiber, vitamins, and minerals due to the refining process of wheat flour [5]. Composite flour, comprised of a blend of different grains or cereals, has garnered attention in scientific research and food product development for its tailored nutritional and functional attributes. Recent studies on composite flour have demonstrated that it offers higher concentrations of proteins, minerals, vitamins, fibers, and phytochemicals compared to single-cereal flour [6].

The banana (*Musa acuminata × balbisiana* (ABB Group)) is a widely cultivated fruit in tropical regions and is important in global agriculture. It has versatile qualities that make it a promising candidate for the development of new food products [7]. Green banana flour (GBF) is made from unripe bananas and is rich in essential elements like potassium, phosphorus, iron, and calcium. It also contains phenolic acids, minerals, vitamins, and resistant starch, which all contribute to promoting good health [8]. GBF contains a high amount of resistant starch, which is not digested in the small intestine but reaches the large intestine and acts as a prebiotic. Prebiotics promote the growth and activity of beneficial gut bacteria, such as *Lactobacilli* spp. Incorporating foods rich in resistant starch, like GBF, into one’s diet can support a healthy gut microbiome and offer various health benefits, including enhanced immune function, digestion, and blood sugar control. GBF is a gluten-free alternative flour that can be used in baking, smoothies, and as a thickening agent. The amount of resistant starch in GBF may vary depending on the ripeness of the bananas used [9]. Green bananas have been found to have potential health benefits due to their indigestible components, according to several studies. Recent scientific inquiries have focused on enhancing the nutritional characteristics of food products by incorporating GBF. Recent studies show that adding GBF to noodles significantly raised levels of resistant starch, total dietary fiber, and vital minerals including magnesium, calcium, phosphorus, and potassium [10].

The pseudo-cereal foxtail millet (*Setaria italica L*.) is gluten-free and cultivated across Asia, Australia, Africa, and South America, particularly in countries like India, China, Niger, and Nigeria. Foxtail millet (FM) is known for its excellent characteristics, including C4 photosynthesis, which is crucial for capturing carbon. Recently, FM has emerged as a viable model for studying plant architecture, drought tolerance, and C4 photosynthesis in grain and bioenergy crops. In addition, the nutritional value of FM is also well-known; its grains have high protein (8.98–14.37%), fat (2.79 to 4.16%), crude fiber (1.34–2.31%), carbohydrates (70.90–75.63%), dietary fiber (14–19%), minerals (1.08–1.57%), vitamin E, folic acid, selenium, carotenoids, and phytochemicals like polyphenol and flavonoids. Compared to wheat and rice, foxtail millet flour (FMF) emerges as a superior choice [11–13]. Regular consumption of millets has been linked to a lower risk of degenerative diseases due to their phytonutrients and bioactive compounds. Additionally, millets offer health advantages such as antioxidant, anti-ulcerative, hypoglycemic, and anti-inflammatory properties [14, 15]. The carbohydrates in millets have lower starch digestibility, moderating blood glucose levels due to slower absorption kinetics [16]. Given their phytochemical profile and potential health benefits, millets are being explored as functional ingredients in various food products, including biscuits, noodles, bread, and beverages [12].

Traditional noodle production predominantly relies on wheat flour, which while providing certain nutritional benefits but its lacks of the diverse array of nutrients and prebiotic properties. Despite the known health benefits of incorporating more diverse ingredients into diets, there is limited research on the use of prebiotic and nutrient-rich FMF and GBF in noodle production. This study addresses the research gap by exploring the novel incorporation of FMF and GBF into noodle production. The objective of this research is to explore the feasibility of developing noodles that are rich in fiber and nutritionally superior by substituting wheat flour with FMF and GBF. This study conducts a thorough evaluation of the effects of FMF and GBF on various characteristics of noodles, including their physiochemical properties, prebiotic potential, antioxidant properties, sensory attributes, and cooking behaviour. By providing detailed insights into these aspects, the study contributes to the development of functional foods that not only meet nutritional needs but also appeal to consumer preferences. This innovative approach could pave the way for healthier noodle products, offering a new direction for the food industry and contributing to public health nutrition.

## Materials and methods

### Reagents and chemicals

The MRS agar; Hi-media, (India) and chemicals used, including gallic acid, DPPH (2,2-diphenyl-1-picrylhydrazyl), Folin–Ciocalteu’s (FC) reagent, ascorbic acid, phosphate buffer, trichloroacetic acid, potassium ferrocyanide, ferric chloride, sodium hydroxide (NaOH), sulfuric acid (H_2_SO4), methanol, potassium acetate, sodium carbonate, and aluminum nitrate, were sourced from Sigma–Aldrich and Merck (Germany).

### Raw materials

The wheat flour (WF), FM (variety: Bari Kaon-2) and GB ((*Musa acuminata × balbisiana* (ABB Group)) were purchased at the neighbourhood market. A 60 mesh (250 μm) sieve was used to filter the WF. Additionally, salt and corn starch were purchased from a nearby market and utilized without further treatment. The same brand and batch of the flour, corn starch and salt were used thought out the study.

### Preparation of FMF

The FM grain was cleaned, washed, and then dried for eight hours at 60°C in an oven (Memmert D-91126, Memmert, Germany). Using an electric grinder, the flour was then finely ground. To obtain a smooth and fine powder, the flour was sieved through a 250-micron (μm) sieve. The FMF was kept at room temperature for further analysis in sealed containers.

### Preparation of GBF

The GB were cleaned and peeled. It is then cut into pieces, blanched in boiling water for 5 minutes, and cooled. The GB slice underwent dehydration in a Memmert D-91126 oven, manufactured by Memmert in Germany, for a duration of 8 hours at a temperature of 60°C. After drying, fine flour (250 μm) was prepared with a grinder and stored air tight in a food-grade polythene bag in a refrigerator until use.

### Preparation of noodles

Noodle-preparing ingredients, such as WF, FMF, GBF, corn starch, iodized salt, and water, were weighed according to Table 1. After manually mixing all the ingredients with warm water, the noodle dough was kneaded for 10 minutes and rested for 20 minutes. A noodle-making machine converts dough into a smooth sheet. After 30 minutes of rest, the noodles were made by the noodle-making machine. The noodles were oven-dried for 8 hours at 68°C. The cool and dry noodles were stored in sealed food-grade polyethylene bags until use.

**Table 1:** Formulation of the experimental noodles.

Ingredients	N0	N1	N2	N3	N4
WF (%)	100	80	70	60	50
FMF (%)	0	10	20	30	40
GBF (%)	0	10	10	10	10
Salt (%)	1.50	1.50	1.50	1.50	1.50
Corn starch (%)	1.60	1.60	1.60	1.60	1.60

Here, N0 = 100% WF; N1 = 80% WF + 10% GBF + 10% FMF; N2 = 70% WF + 10% GBF + 20% FMF; N3 = 60% WF + 10% GBF + 30% FMF; N4 = 50% WF + 10% GBF + 40% FMF

### Probiotic potential analysis

#### Probiotic strains

The probiotic bacterial strains were obtained from the Industrial Microbiology Laboratory’s stock culture collection of the Institute of Food Science and Technology (IFST), Bangladesh Council of Scientific and Industrial Research (BCSIR). The bacterial cultures were kept at -20°C in a 20% (v/v) glycerol solution. The present investigation utilized three bacterial isolates: *Lactobacillus plantarum*, *Lactobacillus rhamnosus*, and *Lactobacillus acidophilus*. The probiotic bacteria were cultured in De Man-Rogosa-Sharpe agar medium for 48 hours at 37°C under anaerobic conditions to activate them.

#### Sample preparation for probiotic bacterial growth

The probiotic bacterial strains were prepared for inoculation by suspending the bacteria in sterile water at a concentration of 10^8^ CFU/mL (0.5 McFarland standard). The bacteria were then diluted in a series of steps (1:100), resulting in a concentration of roughly 1 × 10^6^ CFU/mL as assessed by a colony count test. The MRS broth media supplemented with 10% GBF, 10% FMF and 10% N3 (prepared without salt and with salt) sample was separately prepared and maintained. In addition, a small portion (10%) of GBF, FMF, and N3 noodles was used to study the development of probiotic bacterial strains, taking into account the optimal level to prevent the turbidity of the MRS broth. The MRS medium was used as a control, while the MRS medium and the bacterial strain were used as positive controls. During this study, an experiment was conducted using N3 noodles without salt to investigate the impact of the phytochemical component on the growth of probiotic bacteria. It may be that salt can inhibit or reduce bacterial growth. The MRS broth (1000 μL) media (four different types) were then inoculated with bacterial suspension (100 μL) and incubated at 37°C with anaerobic condition. The optical density was measured at 600 nm for each culture at the following times: 0, 24, 48, 72, and 96 hours [17]. Following this, the specific growth rates of the probiotics in media containing only MRS were determined and compared to the growth curve plots of the probiotic standards, which were expressed in cfu/mL and optical density (OD, λ = 600 nm). The probiotics’ growth plots were used to derive exponential phase growth.

### Nutritional composition

The protein (N × 6.25), crude fiber, fat, ash and moisture content were determined using established AOAC [18] standard methods (methods 979.09, 920.85, 962.09, 923.03, and 925.09 respectively). The amount of carbohydrates was estimated by subtracting the values of the other proximate constituents from 100. The noodles’ energy content (Kcal) was determined using the following equation [19].


$$Total\;energy\;\left(\frac{Kcal}{100g}\right)=[\left(\%\;carbohydrate\times 4)+(\%\;protein\times 4)+(\%\;fat\times 9)+(\%\;dietary\;fiber\times 2\right)]$$ (1)

### Mineral composition

Mineral content was determined using standard analytical methods AOAC [18]. The concentrations of sodium and potassium were determined using a flame photometer (PFP7, Jenway, Germany), while the levels of calcium, magnesium, zinc, iron, copper, and manganese were analysed using an atomic absorption spectrophotometer (ICS-3000, Thermo, USA).

### Color intensity

The noodles, which weighed around 15 grams, were ground into powder using a pestle and mortar. Subsequently, the color of the powdered noodles was measured using a colorimeter (Konica Minolta CR-400, Tokyo, Japan) on the CIE 1976 L*a*b* color scale. This color space includes parameters such as lightness (L*), red-green balance (a*), and yellow-blue balance (b*), which allow for a semi-quantitative expression of the colors [20].

### Cooking quality of noodles

#### Cooking loss

The cooking loss of samples (noodle) was calculated using the AACC [21] method with minor modifications. To evaluate cooking loss, 20 g sample was cooked in boiling water (250 mL) for 10 minutes. The cooking loss of the noodles was calculated using the following equation:

$$Cooking\;loss\;(\%)=\frac{W_{R}}{W_{B}}\times 100\;$$ (2)

Where, W_R_ is the weight of dried residue in cooking water and W_B_ is the initial weight of the noodles.

#### Water uptake

The water uptake was calculated by comparing the weight difference between the uncooked and cooked noodles and expressing it as a percentage. The water uptake was determined using the following equations:

$$Water\;uptake\;(\%)=\frac{W_{A}-W_{B}}{W_{B}}\times 100\;$$ (3)

Where W_B_ = initial weight (noodles) and W_A_ = cooked noodles weight.

#### Cooking yield

The AACC [21] approach was used to evaluate the noodle cooking yield. Using a covered beaker, around 20 g sample (noodle) were carefully measured and cooked for 10 minutes in boiling distilled water (250 mL). The below equation was used to ascertain the noodle cooking yield:

$$Cooking\;yield\;(\%)=\frac{W_{A}}{W_{B}}\times 100$$ (4)

Where W_B_ = initial weight (noodles) and W_A_ = cooked noodles weight.

### Water activity (a_w_) determination

The a_w_ of powdered noodles (2.5 g) was measured using a water activity meter (Novasina RS 200, Axair Ltd., Pfaffikon, Switzerland). The measurement was performed within a hermetically sealed measuring chamber. The tests were conducted at a temperature of 25°C via the chilled-mirror dew point technique. Three replicates of milled noodles were used to test each composition [20].

### Total dietary fiber (TDF) content

The TDF contents of the samples were determined by enzymatic gravimetric (Megazyme-K-TDFR-200A) MES-TRIS buffer AOAC (2005) method-991.43 [18]. The TDF was calculated using the formula:

$$Dietary\;fiber\;(\%)=\frac{(\frac{R_{1\;}+R_{2}}{2})-p-A-B}{\frac{m_{1}+m_{2}}{2}}\times 100$$ (5)

Where: R_1_  =  residue weight 1 from m_1_; R_2_  =  residue weight 2 from m_2_,

m_1_  =  sample weight 1; m_2_  =  sample weight 2,

A  =  ash weight from R_1_; p  =  protein weight from R_2_ and

$$B=blank=\frac{{BR}_{1}+{BR}_{2}}{2}-BP-BA$$

Where: BR  =  blank residue,

BP  =  blank protein from BR_1_,

BA  =  blank ash from BR_2_.

### Antioxidant properties

Phytochemicals were extracted from the noodle samples by method of Kumar et al. [22] with slight changes. Concisely, noodles powder samples (2.0 g) were mixed with 20 mL of methanol and the mixture was placed in a shaking incubator at 25°C for 8 hour. The mixture was centrifuged at 5000 rpm for 10 min and the supernatant were pooled and stored at 4°C used for antioxidant activity using various assays, including the DPPH free radical and FRAPS assay, total phenolic content, and total antioxidant content.

#### DPPH free radical assay

The radical-scavenging activity of DPPH was determined following the method described by Gulcin and Alwasel [23] with slight modifications. Initially 1 mL of sample extract was combined with 2 mL of 0.1 mM DPPH solution in a falcon tube. The falcon tube was then incubated in the dark for 30 minutes. 1 mL of methanol and 2 mL of DPPH were used to make a blank. Methanol was used as a reference. The absorbance was then measured at 517nm. The following formula was used to calculate radical-scavenging activity:

$$DPPH\;radical\;scavenging\;activity\;(\%)=\frac{A_{0}-A_{s}}{A_{0}}\times 100$$ (6)

Where, A_0_ = Absorbance of control and A_S_ = Absorbance of sample

This formula enabled the quantification of the percentage of DPPH radicals scavenged by the sample extract, thereby indicating its antioxidant capacity.

#### Ferric reducing antioxidant power (FRAP) assay

The FRAP test is a widely utilized technique based on electron transfer (ET) mechanisms, which evaluates the capacity of antioxidants to reduce a Fe^3+^ ligand complex to a Fe^2+^ (blue-colored) under acidic conditions. The FRAP assay was conducted on noodle extracts following the method described by Zamankhani et al. [24] with some modifications. In this procedure, 2.5 mL of phosphate buffer solution (0.2M, pH 6.6) were mixed with 2.5 mL of noodle extracts and 2.5 mL of potassium ferricyanide (1%, w/v) in a test tube. After that, the mixture was kept at 50°C for 20 minutes to incubate. Once the incubation period was over, 2.5 mL of a 10% TCA solution was added to every tube. The tubes were then centrifuged at 3000 rpm for duration of 10 minutes. The supernatant obtained after centrifugation was then combined with 2.5 mL of deionized water in individual test tubes. To this mixture, 0.5 mL of 0.1% w/v FeCl_3_ solution was added, and the absorbance was measured at a wavelength of 700 nm using a UV spectrophotometer (Brand: Analytik Jena, Model: Specord 205, Origin: UK). The percent inhibition was calculated using the formula:

$$Inhibition\;(\%)=\frac{A_{s}-A_{0}}{A_{s}}\times 100\;$$ (7)

Where, A_S_ = Absorbance of sample and A_0_ = Absorbance of control sample

The percentage inhibition, which shows the antioxidant activity of the noodle extracts, was calculated using this method.

#### Total phenolic content (TPC)

The TPC was determination using the FC technique with slight modifications [25, 26]. In this procedure, 0.5 mL of the noodle extracts were combined with 1 mL of FC reagent, 4.5 mL of distilled water, and 1 mL of 7.5% sodium carbonate solution. The mixture was left to sit in a dark environment for 30 minutes at a temperature of 25°C. The absorbance was determined at a wavelength of 765 nm. TPC was calculated using a standard gallic acid curve and reported as mg GA eq/100 g of sample. This procedure enabled the quantification of polyphenolic compounds present in the noodle extracts, providing valuable information about their antioxidant potential and nutritional quality.

#### Total antioxidant content (TAC)

The TAC was measured following the technique of Abdullah et al. [27] with slight modifications. Initially, 0.5 mL of extracts were added to a falcon tube containing 5 mL of a reagent solution composed of 4 mM ammonium molybdate, 28 mM sodium phosphate, and 0.6 M sulfuric acid. This resulted in a total volume of 5.5 mL. Subsequently, the tubes were securely sealed and placed in an incubator at 95°C for duration of 90 minutes to facilitate the reaction. The absorbance was measured at 695 nm using a spectrophotometer against a blank after the sample was cooled down. The TAC was determined using the standard ascorbic acid calibration curve and reported as mg AA eq/100 g of sample. This method allowed for the assessment of the overall antioxidant capacity of the noodle extracts, providing valuable insights into their potential health-promoting properties.

### Sensory attributes

A sensory evaluation of cooked noodles was conducted by eleven trained panelists (five males, six females, and aged 21–55 years) from the IFST of the BCSIR. All the panelists selected were individuals who did not smoke. Every participant was situated in their own sensory booth, where the examination took place. The panel’s leader assigned codes to the samples for identification purposes. A sensory test evaluation form was given to each participant, who was then instructed to provide feedback on various quality aspects such as color, texture, flavor, taste, mouthfeel, and overall acceptance. Furthermore, the panelists were asked about the general acceptability of the noodles in the evaluation form, which was divided into separate sections. The quality of the samples was evaluated using a 9-point hedonic scale, ranging from highly liked (9) to highly disliked (1) [28]. Every participant provided their signed informed consent on the same form during the evaluation. This study has been approved for human subject participation by the IFST, BCSIR, Dhaka-1205, Bangladesh, considering food products developed from generally recognized as safe (GRAS) materials. The report presents the average ± SD of the sensory test, as determined by the head of panelists.

### Statistical analysis

Data were presented as mean ± SD for three replicates. Graphical presentations were created using Origin 2018 software. Statistical analysis of variance (one-way ANOVA) was performed using SPSS (version 22.0, IBM, Chicago, IL, USA) to determine statistically significant differences (p < 0.05) between means.

## Results and discussion

### In vitro prebiotic potential analysis

**Fig 1:**
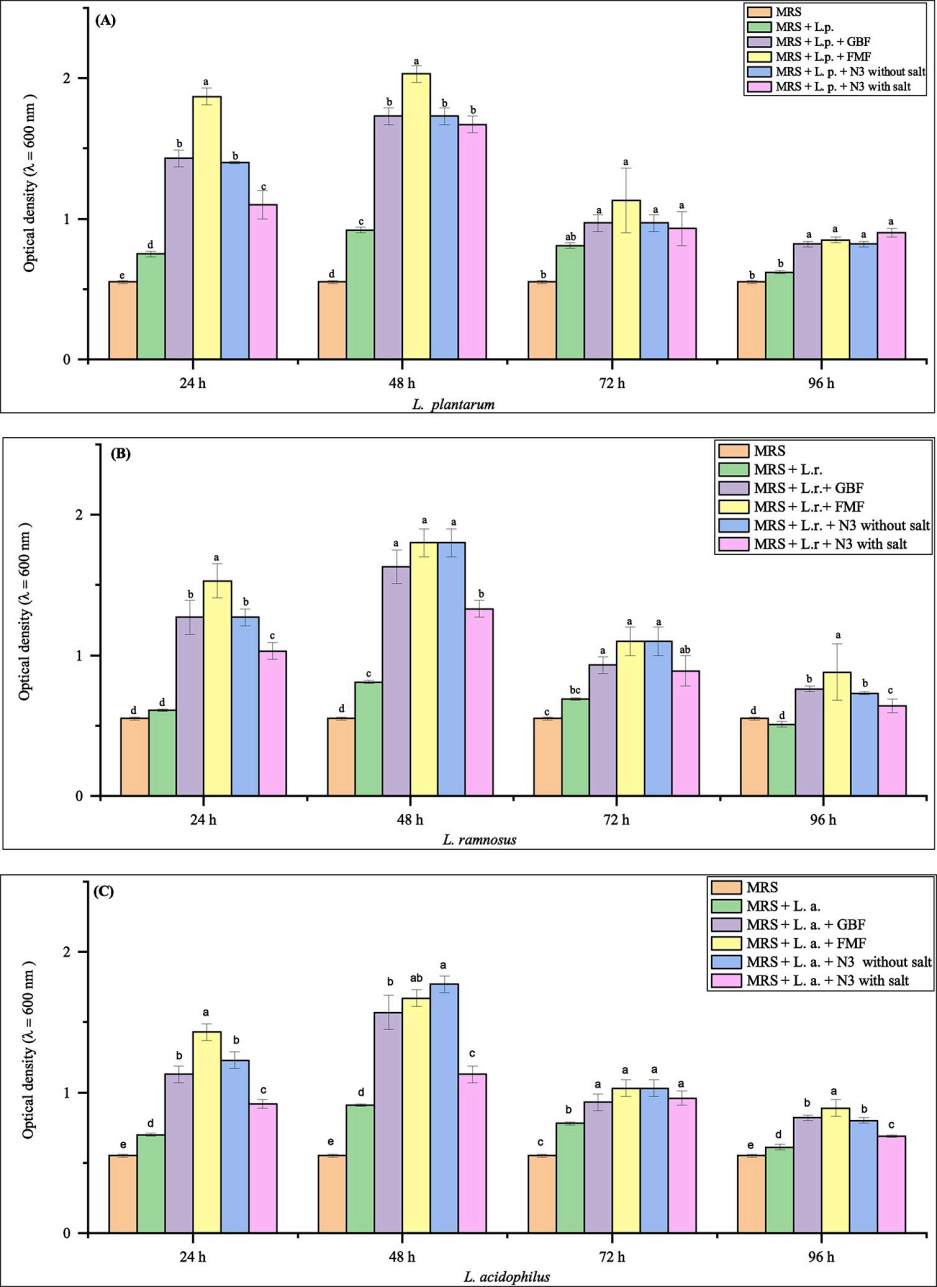
The growth kinetics of probiotics bacteria in MRS medium supplemented with prebiotics. (A) *L*. *plantarum*, (B) *L*. *rhamnosus* and (C) *L*. *acidophilus*. Values are expressed as mean ± SD (n  =  3). Statistical analysis performed ANOVA followed by Duncan’s multiple range tests. Different lowercase letters above the column indicate significant differences (p < 0.05). Here, L. p. = *Lactobacillus plantarum;* L.r. *= Lactobacillus rhamnosus;* L. a. *= Lactobacillus acidophilus;* GBF = Green banana flour (10%); FMF = Foxtail millet flour (10%) and N3 = 30% FMF + 10% GBF + 60% WF noodles.

Prebiotics are indigestible dietary components that actively foster the growth of beneficial bacteria or enhance the activity of bacteria that promote good health. As consequence, they are regarded to be important functional foods. However, prebiotics may also enhance the presence and efficacy of ingested probiotic microorganisms. According to the International Scientific Association of Probiotics and Prebiotics, "prebiotics" refers to a substance that is specifically broken down by the microorganisms in the gut and provides health advantages to the host [29, 30]. This research aimed to optimize prebiotic capability to enhance the growth of representative probiotic microorganisms, including *L*. *plantarum*, *L*. *rhamnosus*, and *L*. *acidophilus*. The present study assessed four different types of samples e.g., 10% GBF, 10% FMF, 10% N3 (without salt), and 10% N3 (with salt), for in vitro prebiotic potential. The studies have demonstrated that the tested three probiotic bacteria thrive when supplemented with FMF and GBF. The growth rate of probiotic bacterial species in relation to different samples is illustrated in Fig 1. This result is very much consistent with the results of Guadalupe Monserrat Alvarado-Jasso et al., 2020 [31]. Similarly, the result indicated that 10% FMF significantly increased the growth of probiotic bacterial species within 48 hours at 37°C under anaerobic conditions. Among the three probiotic bacteria, the highest prebiotic activity was achieved for *L*. *plantarum* (OD_600nm_ = 2.03 ± 0.06). The experimental findings reveal a logarithmic reduction in the quantity of probiotic bacteria from 72 to 96 hours that is statistically significant (p < 0.05). This indicates that the growth curve has reached a stationary phase. A combination of 10% GBF and 10% FMF clearly promotes the development of probiotic strains in both monocultures, indicating the combination also has the greatest potential as a good prebiotic source. Though the noodles samples are prepared by the assembly of both FMF and GBF, the N3 sample also exhibits potential prebiotic capability. Out of the three probiotic isolates tested, the N3 sample showed the most substantial increase in *L*. *acidophilus*-specific growth rates. The strains *L*. *plantarum*, *L*. *rhamnosus* and *L*. *acidophilus* were used to represent typical probiotic strains since they are mostly found in the colon and small intestine, respectively. Numerous clinical studies have delved into the impact of specific strains on energy metabolism and gut microbiota in obese mice, as well as their potential to enhance the human gut microbiota [32]. All three examined probiotics were able to proliferate on medium supplemented with the investigated prebiotics, suggesting that a suitable proportion of mixed prebiotics could be utilized as a functional food constituent. Similar studies have documented the identical growth rate specifications as the current investigation [33]. The previous study by Baek et al. [17] indicated the similar probiotic bacterial growth on GBF. Regarding the growth and quantity of probiotic bacteria, no statistically significant (p < 0.05) variations were identified in response to 10% N3 noodles without salt and N3 with salt. The previous study by Powthong et al. [29] describes the similar result to the current study. The significant increase in the growth rate of probiotic bacteria indicated the samples’ prebiotic capability.

Foxtail millet and green banana flour have prebiotic activity due to their substantial amounts of dietary fiber and phytochemicals, both of which are important in improving gut health and creating an environment conducive to good gut flora. The dietary fiber found in foxtail millet and green bananas is a combination of soluble and insoluble fibres, including pectins, resistant starch, and cellulose. Once these fibers make their way to the colon, they are broken down through fermentation by the gut microbiota. The fermentation process encourages the growth of helpful bacteria like Bifidobacteria and Lactobacilli. Microorganisms ferment these fibres, converting them into short-chain fatty acids (SCFAs) such as acetate, propionate, and butyrate, which are known for their multiple health benefits, including colon health, inflammation reduction, and overall gut health. Butyrate, for instance, serves as the primary source of energy for the cells lining the colon, playing an important role in gut health [17, 34, 35]. On the other hand, several studies have demonstrated that foxtail millet and green bananas contain phytochemicals like polyphenols and flavonoids, which have been found to have prebiotic properties. These compounds have the ability to encourage the growth of beneficial bacteria such as Lactobacillus and Bifidobacterium while simultaneously impeding the growth of harmful bacteria. In addition, they also improve the production of mucin, which is a protective layer in the gut, and enhance the function of the gut barrier [36–38].

### Noodles nutrient composition

The nutrient composition of the prepared noodles, including moisture, ash, fat, crude fiber, protein, and carbohydrate content, are summarized in Table 2. FMF and GBF enriched noodles (N1 to N4) exhibited significantly higher moisture content (6.40 ± 0.36 to 8.09 ± 0.13 g/100 g) compared to N0 (5.31 ± 0.13 g/100 g). Additionally, FMF and GBF enriched noodles had significantly (p < 0.05) higher ash content (2.00 ± 0.09 to 2.29 ± 0.05 g/100 g) compared to N0 (1.54 ± 0.06 g/100 g). The ash content of foxtail millet and unripe banana flour is higher compared to wheat flour, resulting in an increase in ash content. Given that ash content serves as an indicator of mineral content, it may be inferred that samples with greater ash content are likely to possess higher mineral content. The fat content of formulated noodles varied from 1.30 ± 0.13 to 2.09 ± 0.13 g/100 g, with N3 and N4 noodles exhibiting significantly (p < 0.05) higher fat content compared to N0 (1.23 ± 0.17 g/100 g). Due to the different ratios of wheat and foxtail millet flour used in noodle production, the fat content in each sample may vary. Formulated noodles also demonstrated significantly (p < 0.05) higher crude fiber content (0.49 ± 0.03 to 1.14 ± 0.18 g/100 g) compared to N0 (0.18 ± 0.03 g/100 g). The N0 had the lowest amount of protein (11.80 ± 0.10 g/100 g), whereas N4 noodles had a considerably higher amount of protein (12.10 ± 0.11 g/100 g) because of the use of foxtail millet flour. Moreover, the FMB noodles contained significantly lower (p < 0.05) amount of carbohydrates (79.18 ± 0.44 to 75.52 ± 0.38 g/100 g) than the N0 (80.12 ± 0.46 g/100 g). Carbohydrates are organic molecules that provide energy. The low carbohydrate content of FMB noodles provides various health benefits, including enhanced colon digestion and a reduction in constipation. [1]. The study conducted by Arora et al. [39] revealed that the incorporation of foxtail millet into food formulations led to a notable enhancement in the protein, fat, and fiber composition, while concurrently exhibiting a decrease in the carbohydrate content. According to Kumari et al. [40] the replacement of wheat flour with foxtail millet flour had a direct impact on the moisture, fat, ash, and protein contents of the composite flours. Particularly, when the substitution level grew from 10% to 40%, these contents also increased. The inclusion of proteins, dietary fiber, fat, and minerals from wheat flour enhanced the nutritional content of composite flours.

**Table 2:** Nutrient and mineral composition of noodles.

Nutrients	N0	N1	N2	N3	N4
Moisture (g/100 g)	5.31 ± 0.13^c^	5.68 ± 0.20^c^	6.40 ± 0.36^b^	7.80 ± 0.11^a^	8.09 ± 0.13^a^
Ash (g/100 g)	1.54 ± 0.06^c^	2.00 ± 0.09^b^	2.10 ± 0.11^ba^	2.20 ± 0.06^ba^	2.29 ± 0.05^a^
Protein (g/100 g)	11.80 ± 0.10^a^	11.84 ± 0.11^a^	11.90 ± 0.18^a^	11.95 ± 0.17^a^	12.10 ± 0.11^a^
Fat (g/100 g)	1.23 ± 0.17^c^	1.30 ± 0.13^c^	1.56 ± 0.01^cb^	1.82 ± 0.07^ba^	2.09 ± 0.13^a^
Crude fiber (g/100 g)	0.18 ± 0.03^d^	0.49 ± 0.03^c^	0.71 ± 0.11^bc^	0.92 ± 0.08^ba^	1.14 ± 0.18^a^
Carbohydrate (g/100 g)	80.12 ± 0.46^a^	79.18 ± 0.44^b^	78.05 ± 0.39^b^	76.23 ± 0.33^c^	75.52 ± 0.38^c^
Energy (Kcal/100 g)	378.75 ± 0.16^a^	375.75 ± 0.71^b^	373.79 ± 1.46^b^	369.10 ± 0.25^c^	368.91 ± 0.49^c^
Sodium (mg/100 g)	341.82 ± 7.90^a^	339.89 ± 8.81^a^	339.72 ± 6.64^a^	340.73 ± 9.30^a^	341.12 ± 7.14^a^
Potassium (mg/100 g)	304.43 ± 7.01^a^	315.21 ± 6.07^a^	318.23 ± 8.82^a^	319.89 ± 8.84^a^	321.56 ± 10.22^a^
Calcium (mg/100 g)	148.98 ± 9.72^a^	152.42 ± 8.42^a^	153.62 ± 8.74^a^	155.28 ± 7.44^a^	156.33 ± 7.29^a^
Magnesium (mg/100 g)	40.89 ± 6.73^b^	60.21 ± 6.45^a^	63.57 ± 7.99^a^	66.35 ± 5.70^a^	71.01 ± 5.81^a^
Iron (mg/100 g)	2.33 ± 0.10^d^	2.97 ± 0.11^c^	3.59 ± 0.17^b^	3.73 ± 0.17^b^	4.34 ± 0.18^a^
Copper (mg/100 g)	0.67 ± 0.05^c^	3.52 ± 0.22^b^	3.88 ± 0.26^ba^	4.04 ± 0.28^ba^	4.37 ± 0.18^a^
Zinc (mg/100 g)	0.44 ± 0.07^a^	0.48 ± 0.09^a^	0.52 ± 0.10^a^	0.55 ± 0.09^a^	0.59 ± 0.09^a^
Manganese (mg/100 g)	0.86 ± 0.07^c^	2.67 ± 0.16^b^	2.78 ± 0.18^b^	3.07 ± 0.20^b^	3.85 ± 0.16^a^
Dietary Fiber (g/100 g)	7.33 ± 0.13^e^	9.37 ± 0.14^d^	10.96 ± 0.16^c^	11.88 ± 0.11^b^	12.71 ± 0.12^a^

Here, N0 = 100% WF; N1 = 80% WF + 10% GBF + 10% FMF; N2 = 70% WF + 10% GBF + 20% FMF; N3 = 60% WF + 10% GBF + 30% FMF; N4 = 50% WF + 10% GBF + 40% FMF

Values are means of triplicates ± standard deviation. Statistical analysis performed ANOVA followed by Duncan’s multiple range tests. The mean values with different uppercase letters in each row are significantly (p < 0.05) different.

### Mineral composition of noodles

Minerals have a significant impact on several biological processes inside the human body. The primary role of sodium is to regulate the quantity and allocation of water inside our bodies, which helps to control our blood pressure. Potassium is necessary for the processes of protein synthesis and glucose metabolism, as well as for regulating the heartbeat, muscles, and neurons. Calcium is a vital constituent of skeletal structures, such as bones and teeth, and plays a crucial role in the prevention of osteoporosis [41]. As can be seen from Table 2, sodium, potassium, and calcium content per 100 g sample of prepared noodles were 339.72 ± 6.64 to 341.82 ± 7.90 mg/100 g, 304.43 ± 7.01 to 321.56 ± 10.22 mg/100 g, and 148.98 ± 9.72 to 156.33 ± 7.29 mg/100 g, respectively.

Magnesium serves as a cofactor in more than 300 enzyme systems that oversee many biochemical functions in the body, such as protein synthesis, muscle and neuron function, blood glucose management, and blood pressure control. Magnesium facilitates the movement of calcium and potassium ions across cell membranes [42]. The FMB noodles contained 60.21 ± 6.45 to 71.01 ± 5.81 mg/100 g of magnesium which were significantly (p < 0.05) higher than N0 (40.89 ± 6.73 mg/100 g). The iron content of FMB noodles (2.97 ± 0.11 to 4.34 ± 0.18 mg/100 g) were significantly (p < 0.05) higher than N0 (2.33 ± 0.10 mg/100 g). Iron is necessary for the synthesis of hemoglobin (an oxygen carrier in red blood cells), myoglobin, and enzymes and coenzymes. Copper is an essential mineral that serves a multitude of vital functions within the human body, such as supporting connective tissue and blood vessels. It additionally supports brain development and helps to maintain immune systems [41]. The copper content of FMB noodles were 3.52 ± 0.22 to 4.37 ± 0.18 mg/100 g, which has the significantly (p < 0.05) higher than N0 (0.67 ± 0.05 mg/100 g). Manganese and zinc are important co-factors that are found in certain enzymes. The FMB noodles contained 2.67 ± 0.16 to 3.85 ± 0.16 mg/100 g of manganese, which was significantly (p < 0.05) higher than N0 (0.86 ± 0.07 mg/100 g). The zinc contents of the prepared noodles were 0.44 ± 0.07to 0.59 ± 0.09 mg/100 g sample. The incorporation of 30% FMF and 10% GBF into noodles (N3) resulted in enhanced mineral content, contributing to the nutritional value of the product. Meherunnahar et al. [1] reported that FTM flour impacted the noodle’s mineral composition. The noodles’ sodium, potassium, calcium, and iron content increased with the addition of FTM flour. Amini Khoozani et al. [43] observed a rise in the levels of sodium, potassium, magnesium, and calcium in bread that included 10% to 30% whole green banana flour (WGBF).

### Color intensity of noodles

**Fig 2:**
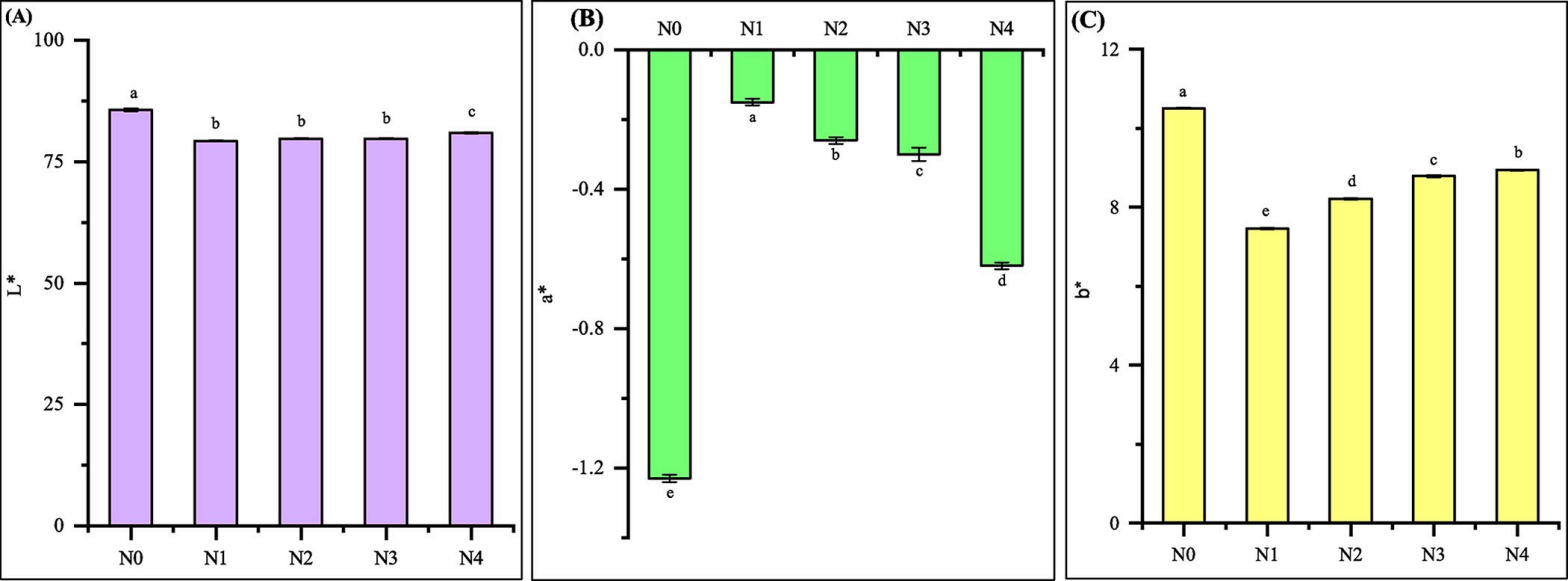
Color intensity: (A) L*, (B) a* and (C) b* of noodles. Values are expressed as mean ± SD (n  =  3). Statistical analysis performed ANOVA followed by Duncan’s multiple range tests. Different lowercase letters with the column indicate significant differences (p < 0.05); Here, N0 = 100% WF; N1 = 80% WF + 10% GBF + 10% FMF; N2 = 70% WF + 10% GBF + 20% FMF; N3 = 60% WF + 10% GBF + 30% FMF; N4 = 50% WF + 10% GBF + 40% FMF.

The color, intensity, and brightness of a product are essential factors influencing its perceived quality, especially in the case of noodles where consumers visually assess the product before making a purchase decision [44]. The visual attributes related to the hue and intensity of the raw noodles is depicted in Fig 2. Incorporating FMF and GBF into the formulation is expected to result in increased a* values and decreased L* and b* values. The experimental results indicate a significant increase (p < 0.05) in the a* values, along with a decrease in the L* and b* values for FMF and GBF enriched noodles. Overall, all formulated noodles had lower (p < 0.05) lightness (L*) and yellowness (b*) values, along with higher redness values (a*) than the control. The incorporation of 30% FMF and 10% GBF led to a noticeable decrease in the L* values of the noodle samples [1, 45]. Additionally, this inclusion resulted in a reduction in gluten protein concentration and deterioration in the structural integrity of the noodles, contributing to a darker color [20]. Similar effects have been observed with the addition of non-wheat flours, such as soybean and carrot pomace flour, and potato flour in noodle production, leading to decreased L* values [46, 47]. The N3 noodles exhibited significantly (p > 0.05) higher a* values compared to the N0. The pigment compositions of 30% FMF and 10% GBF, in conjunction with noodle processing methods, significantly influenced the noodles’ color. The formulated noodles also showed a significant (p < 0.05) decrease in yellowness (b*) when compared to the control noodles. Incorporating 30% FMF and 10% GBF led to a notable reduction in the b* value, indicating a decrease in the yellow hue typically associated with wheat flour-based noodles. The findings align with the study by [48, 49], which demonstrated an increase in a* values and a decrease in both L* and b* values with the incorporation of GBF into noodles.

### Noodles cooking quality

#### Cooking loss

**Fig 3:**
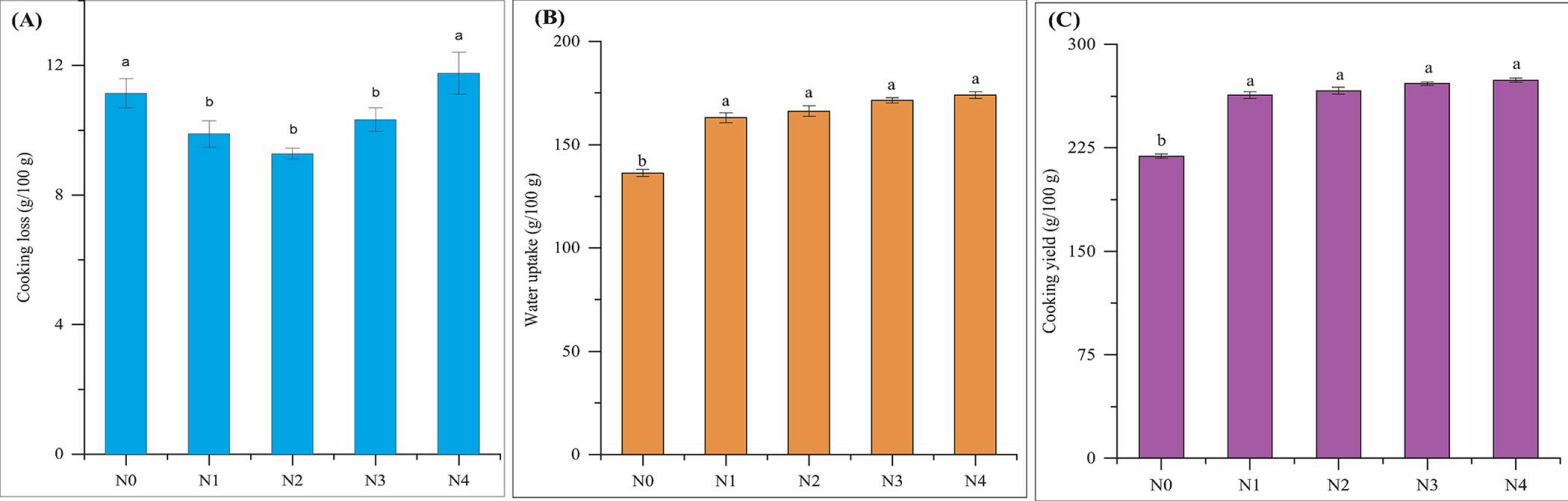
Cooking quality: (A) Cooking loss, (B) Water uptake and (C) Cooking yield of noodles. Values are expressed as mean ± SD (n  =  3). Statistical analysis performed ANOVA followed by Duncan’s multiple range tests. Different lowercase letters above the column indicate significant differences (p < 0.05); Here, N0 = 100% WF; N1 = 80% WF + 10% GBF + 10% FMF; N2 = 70% WF + 10% GBF + 20% FMF; N3 = 60% WF + 10% GBF + 30% FMF; N4 = 50% WF + 10% GBF + 40% FMF.

Cooking loss means the quantity of solid components that dissolve into the cooking water. It is a measure of both the damage suffered by noodles during cooking and their ability to retain their structure in hot water. Consequently, cooking loss is commonly used as an indicator of the overall quality of noodles [20]. For optimal results, noodles should have a cooking loss of no more than 10% to provide the desired cooking quality [50]. In the current research, the cooking loss of all noodles ranged from 9.28 ± 0.17 to 11.76 ± 0.65 g/100 g, indicating favorable cooking quality across the noodle samples. Fig 3A illustrates the cooking loss of all noodle samples. The findings demonstrated a significant (p < 0.05) decrease in cooking loss when the amount of GBF (10%) and FMF (varying from 10 to 30%) in the formulation increased in comparison to the N0. On the other hand, noodles enriched with 10% GBF and 40% FMF exhibit a slight increase in cooking loss when compared to the N0, which is not significant (p > 0.05). These findings suggest that the incorporation of 10% GBF and 10%, 20%, and 30% FMF in noodles resulted in a reduction in cooking loss. This phenomenon may be attributed to the unripe banana and foxtail millet flour’s high capacity for water absorption, which allows for increased water absorption into the gelatinized matrix structures of the noodles [20].

#### Water uptake

The measurement of water uptake was conducted as part of the assessment of cooking quality criteria, and the results are presented in Fig 3B. This measurement indicates the degree of moisture absorption in the noodles, with higher values generally considered unfavorable [51]. The water uptake of noodles prepared from FMF and GBF was notably (p < 0.05) greater than that of N0. Specifically, the water uptakes of FMF and GBF enriched noodles were 163.06 ± 2.45 g/100 g (N1), 166.28 ± 2.54 g/100 g (N2), 171.44 ± 1.26 g/100 g (N3), and 173.97 ± 1.62 g/100 g (N4), while the N0 exhibited a water uptake of 136.23 ± 1.73 g/100 g. The water uptake rate is directly influenced by the gluten protein level in noodles. During the heating process, the gluten protein in the noodles will undergo denaturation and form a bond, preventing water from penetrating at the gelatinization temperature [52]. The enhanced water uptake in noodles made from FMF and GBF can be attributed to the presence of gluten protein and the inclusion of FMF and GBF. The flour components break down the gluten-protein network, making it easier for water to be absorbed. The noodles made with FMF and GBF have a high water absorption capacity, which can be attributed to the presence of high levels of amylose and dietary fiber. These components contribute to a greater ability to retain water compared to wheat flour. Consequently, formulated noodles demonstrated higher water absorption compared to the control noodles. Anggraeni et al. [53] demonstrated the development of dry noodles using unripe banana flour. The investigation of the physiochemical characteristics of noodles incorporating 0, 10, and 30 percent unripe banana flour showed that the water uptake rose to 157, 169, and 180 percent respectively.

#### Cooking yield

Dry noodles have the ability to absorb water during the cooking process, which is known as the cooking yield. Fig 3C illustrates the cooking yield of each noodle sample. The cooking yield of all the FMF and GBF enrich noodles ranged from 263.06 ± 2.45 to 273.97 ± 1.62 g/100 g. It had been seen that the cooking yield of the FMF and GBF-enriched noodles was significantly higher (p < 0.05) than the N0, which had a cooking yield of 218.88 ± 1.66 g/100 g (N0). The incorporation of FMF (10–40%) and GBF (10%) resulted in an increase in the cooking yield of noodles. Research has indicated that FMF and GBF contain a significant amount of dietary fiber, which enables them to effectively absorb and retain water [54]. Noodles with desirable cooking characteristics typically demonstrate minimal cooking loss and substantial cooking yield. Among the noodle samples, N1, N2, and N3 exhibited the lower cooking loss and better cooking yield. These findings suggest that the noodles in sample N3 displayed superior cooking quality compared to all other noodles in the study.

### Water activity of noodles

**Fig 4:**
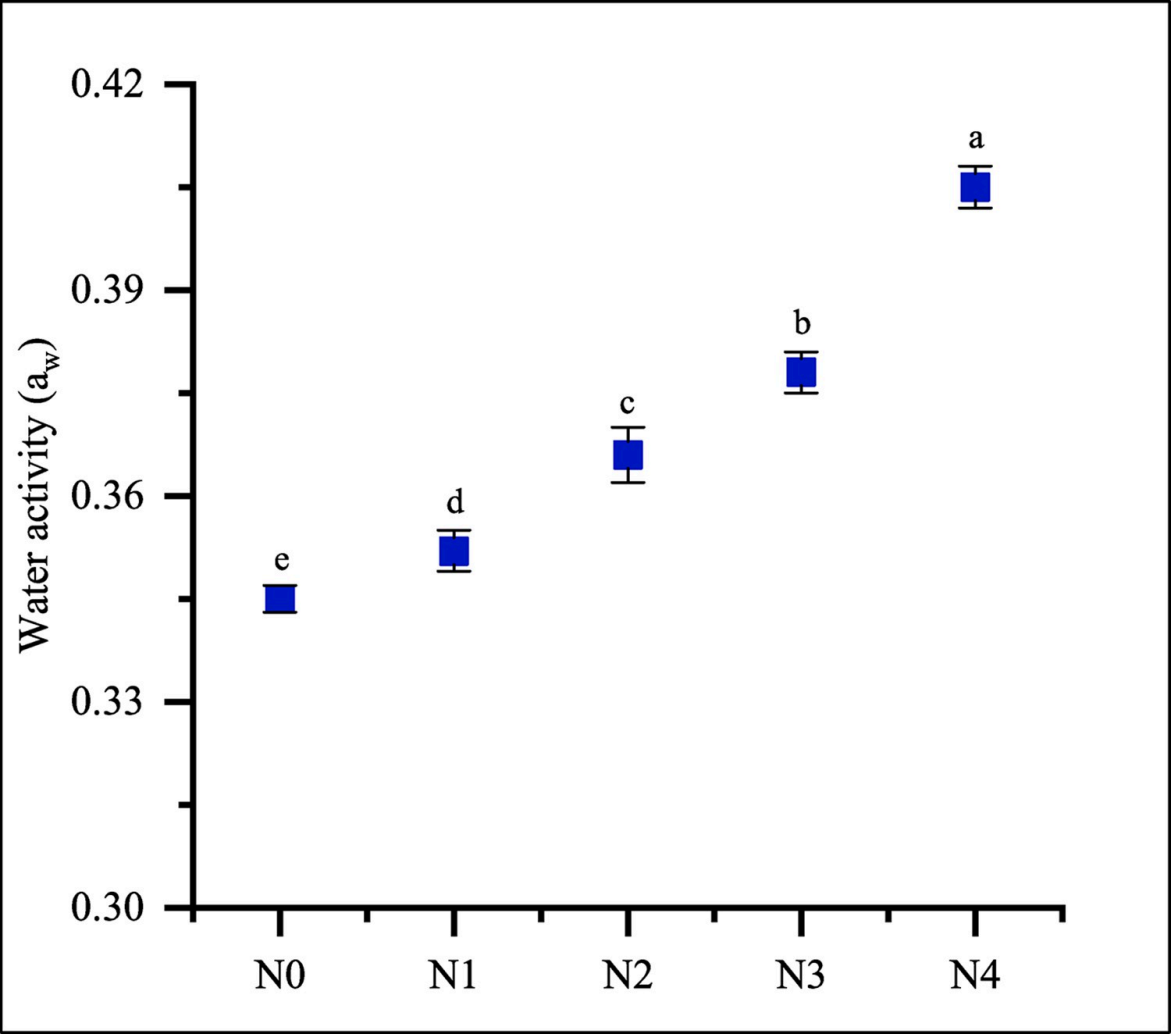
Water activity of noodles. Values are expressed as mean ± SD (n  =  3). Statistical analysis performed ANOVA followed by Duncan’s multiple range tests. Different lowercase letters within the graph indicate significant differences (p < 0.05); Here, N0 = 100% WF; N1 = 80% WF + 10% GBF + 10% FMF; N2 = 70% WF + 10% GBF + 20% FMF; N3 = 60% WF + 10% GBF + 30% FMF; N4 = 50% WF + 10% GBF + 40% FMF.

Water is essential for maintaining the quality and stability of food, as it can impact its composition, mobility, and function. Water activity plays a crucial role in the world of food, as it directly impacts the growth of microorganisms. Proper storage is necessary when it comes to preserving the quality of certain foods, such as fresh noodles with higher moisture content. Microorganisms may be effectively controlled by reducing water activity to low levels. Food products with an a_w_ below 0.61 do not need refrigeration as there is no microbial growth in this environment. However, they should be stored in moisture-resistant packaging to prevent [55]. Fig 4 illustrates the water activity of the prepared noodles. FMF and GBF-enrich noodles (N2, N3, and N4) show significantly (p < 0.05) lower water activity than control noodles. FMF and GBF*-*enriched noodles had a water activity (a_w_) below 0.40, classifying them as microbiologically stable foods. Proper storage in moisture-resistant packaging is essential for maintaining the quality and stability of these noodles, as lower water activity levels inhibit microbial growth [56, 57].

### TDF of noodles

TDF refers to a class of food ingredients that are not absorbed or digested in the small intestine (human) but may ferment partially or completely in the large intestine [58]. It consists of various types of plant-derived carbohydrate polymers, including polysaccharides and oligosaccharides such as resistant starch, inulin, pectin compounds, cellulose, hemicelluloses, and gums. In addition to carbohydrates, it might also include lignin, waxes, phytates, cutin, saponins, resistant proteins, and polyphenols [59, 60]. Dietary fibers are key components of low-calorie products that have gained significant importance recently. Apart from their numerous health benefits, dietary fibers offer technical and functional qualities that can be utilized in food production. They exert important physiological effects on glucose, lipid, and mineral bioavailability, and also regulate processes in the large intestine. Research indicates that dietary fibers may help reduce the risk of several gastrointestinal disorders, including cardiovascular disease, constipation, gastroesophageal reflux disease (GERD), duodenal ulcer, hemorrhoids, stroke, diabetes, obesity, diverticulitis, high blood pressure, and colon cancer [61–64]. The quantity of TDF in the developed noodles is shown in Table 2. There was a noteworthy (p < 0.05) variation seen in the total quantity of TDF in the prepared noodles, with values ranging from 9.37 ± 0.14 g/100 g (N1), 10.96 ± 0.16 g/100 g (N2), 11.88 ± 0.11 g/100 g (N3), and 12.71 ± 0.12 g/100 g (N4), while N0 exhibited 7.33 ± 0.13 g/100 g. In nutritional analysis, it has been noted that both FMF and GBF have higher TDF content compared to regular wheat flour. Therefore, the incorporation of 30% FMF and 10% GBF led to a significant enhancement in the overall TDF content of the formulated noodles. Kumari et al. [40] reported that the composite flour, produced by replacing 30% of rice flour with FMF, had a TDF content of 21.97%, while rice flour alone had a TDF content of only 1.83%. Arora et al. [39] found that the TDF content of FMF and traditional cereal-based products differed statistically significantly (p < 0.05). The foxtail millet-based products exhibited a range of 7.72 to 13.47 g/100 g of TDF, while the control group had a range of 3.82 to 10.32 g/100 g.

### Antioxidant properties of noodles

**Fig 5:**
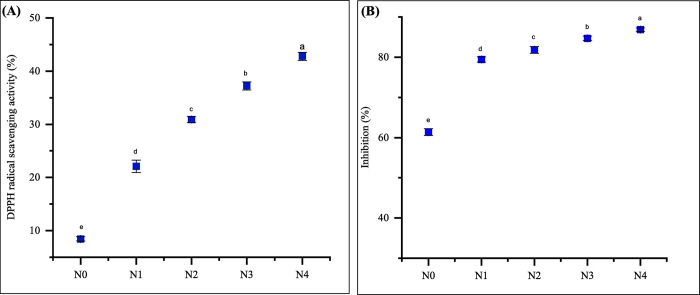
Antioxidant activity: (A) DPPH radical scavenging activity and (B) FRAP assay of noodles extract. Values are expressed as mean ± SD (n  =  3). Statistical analysis performed ANOVA followed by Duncan’s multiple range tests. Different lowercase letters within the graph indicate significant differences (p < 0.05); Here, N0 = 100% WF; N1 = 80% WF + 10% GBF + 10% FMF; N2 = 70% WF + 10% GBF + 20% FMF; N3 = 60% WF + 10% GBF + 30% FMF; N4 = 50% WF + 10% GBF + 40% FMF.

Free radicals are detrimental because they induce oxidative stress. They interact with biological components such as DNA and proteins, potentially resulting in harm to cells. Elevated levels of free radicals have been associated with various chronic conditions affecting the liver, heart, and cancer. FMF and GBF are rich sources of fiber, phytochemicals, and antioxidants. Phytochemicals with antioxidant properties are indispensable for the prevention and treatment of numerous human diseases [65]. Both DPPH and FRAP assays are commonly employed to evaluate the antioxidant activity of wheat-based and functionalized products [66, 67]. The assessment of antioxidant activity was conducted by evaluating the FRAP and DPPH scavenging activity of noodles developed with FMF and GBF enrich noodles, as presented in Fig 5.

### DPPH radical scavenging activity

The DPPH assay is commonly used to assess the potential of chemicals to act as hydrogen donors or free radical scavengers and to evaluate the antioxidant activity of foods and beverages. One of the characteristics of DPPH is that it is violet-colored organic nitrogen radical that is free and stable. When free radicals are scavenged, the color of DPPH changes from violet to light yellow or colorless. In the present study, the antioxidant capacity of the noodles was evaluated by their DPPH free radical scavenging activity (%). Fig 5A illustrates the ability of the noodles to scavenge DPPH-free radicals. The formulated noodles (N1 to N4) showed notably higher (p < 0.05) antioxidant activity against DPPH radicals, ranging from 22.10 ± 1.16% to 42.78 ± 0.77%, compared to the N0, which had an activity of 8.44 ± 0.41%. The antioxidant activity demonstrated that the DPPH radical scavenging capacity increased with increasing amounts of GBF (10%) and FMF. This occurrence may be attributed to the increased concentrations of antioxidant compounds found in foxtail millet and raw banana flour, in comparison to wheat flour. Kumari et al. [40] observed that the incorporation of FMF in rice cookies increased the DPPH scavenging activity of rice cookies. Similarly, Marak et al. [68] showed that cookies containing 30% FMF and 10% GBF had significantly higher DPPH scavenging activity.

#### FRAP assay

In the realm of nutritional research, the FRAP test has gained widespread utility. This test serves to explore the absorption of antioxidants from food and to ascertain the "TAC" of diverse diets [69]. The reducing power assay, a commonly employed method in food science, assesses the capacity of various compounds to facilitate the reduction of Fe^3+^ ions to Fe^2+^ ions. Experimental findings unequivocally demonstrate that all the extracts effectively catalyze the reduction of Fe^3+^ to Fe^2+^ through electron transfer mechanisms. Fig 5B, illustrates the FRAP of the noodle extracts. FRAP analysis findings for the produced noodles ranged from 79.42 ± 0.66% to 86.85 ± 0.38% for samples N1 to N4, with sample N0 at 61.37 ± 0.85%. The analysis revealed that the FRAP scavenging activity of the developed noodles was significantly higher (p < 0.05) when the quantity of FMF and GBF in the formulation was increased.

#### TPC and TAC of noodles

**Fig 6:**
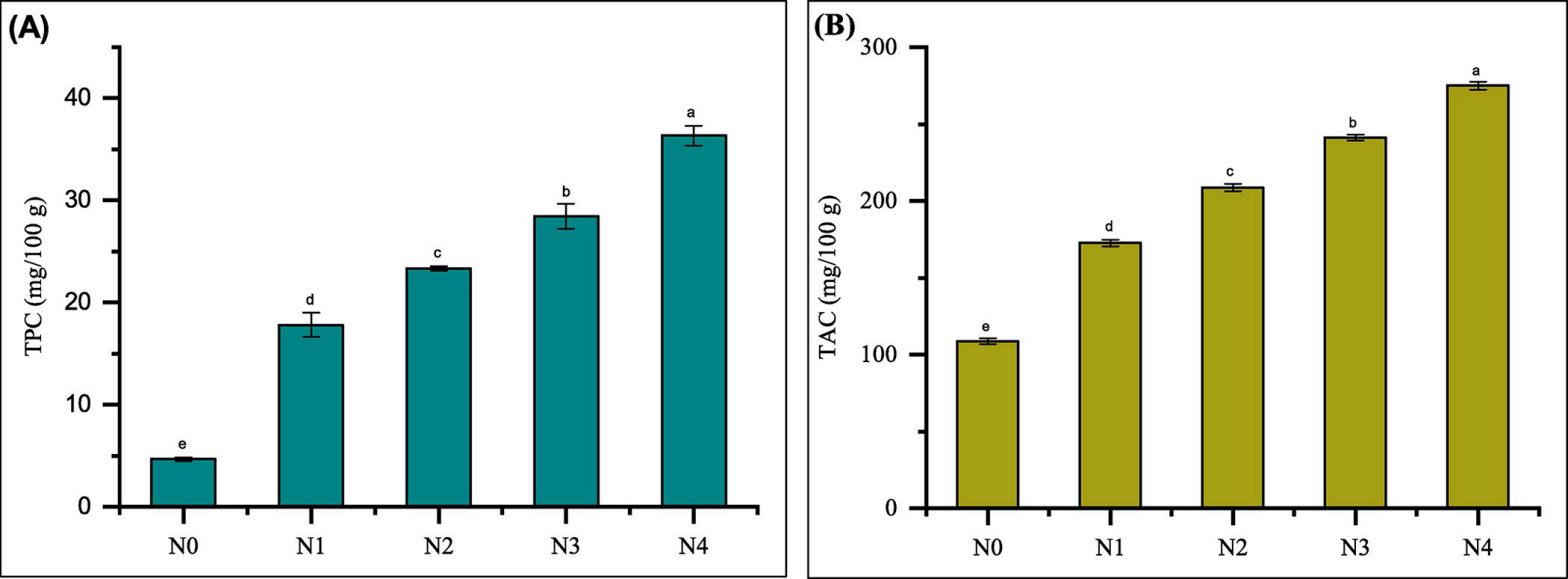
Antioxidant characteristics: (A) Total phenolic content (TPC) and (B) Total antioxidant content (TAC) of noodles. Values are expressed as mean ± SD (n  =  3). Statistical analysis performed ANOVA followed by Duncan’s multiple range tests. Different lowercase letters above the column indicate significant differences (p < 0.05); Here, N0 = 100% WF; N1 = 80% WF + 10% GBF + 10% FMF; N2 = 70% WF + 10% GBF + 20% FMF; N3 = 60% WF + 10% GBF + 30% FMF; N4 = 50% WF + 10% GBF + 40% FMF.

Phenolic compounds are a diverse range of phytochemicals produced by plants as secondary metabolites. They are well-known for the natural antioxidant characteristics that they possess, play an important role in the process of improving the oxidative stability of a wide range of food products and provide considerable health advantages [68]. Because phenolic compounds can form various kinds of interactions, such hydrogen bonds, ionic bonds, and hydrophobic interactions, with the components of the food matrix, their health benefits to humans have been studied. As a result, efforts have been made to maximize the production of phenolic compounds-enriched foods without changing their organoleptic and structural qualities. For example, adding phenolic compounds to food can lower the chance of getting diseases caused by free radicals. However, the food’s qualities may change, leading to a change in texture, taste, or smell, which could be good or bad. Sensory requirements play a crucial role in determining the quality of food, since they directly influence the consumer’s acceptance of the product [70]. The antioxidant properties of noodles can be determined by assessing their TPC and TAC. The results regarding the TPC and TAC of noodles are presented in Fig 6.

The TPC of noodles enriched with FMF and GBF ranged from 17.81 ± 1.19 to 36.35 ± 0.98 mg GA eq/100 g. The TPC values observed in these developed noodles were found to be significantly (p < 0.05) different from those of the N0 (4.66 ± 0.19 mg GA eq/100 g). A similar trend was observed in the TAC of noodles that contained FMF and GBF. The TAC of noodles (N1 to N4) ranged from 172.57 ± 2.08 to 274.94 ± 2.51 mg AA eq/100 g, which was substantially different (p < 0.05) from the TAC of the N0, which was 108.73 ± 1.98 mg AA eq/100 g. The findings correspond with previous research conducted by Kumari et al. [40] and Marak et al. [68], which demonstrated increased TPC and TAC in food products enriched with foxtail millet. Specifically, the addition of FMF to rice cookies and cookies treated with FMF and ginger powder led to substantial increases in both TPC and TAC. These results underscore the potential of incorporating 30% FMF and 10% GBF into noodle formulations to enhance their antioxidant properties, thereby offering consumers products with added health benefits.

### Sensory attributes of noodles

**Fig 7:**
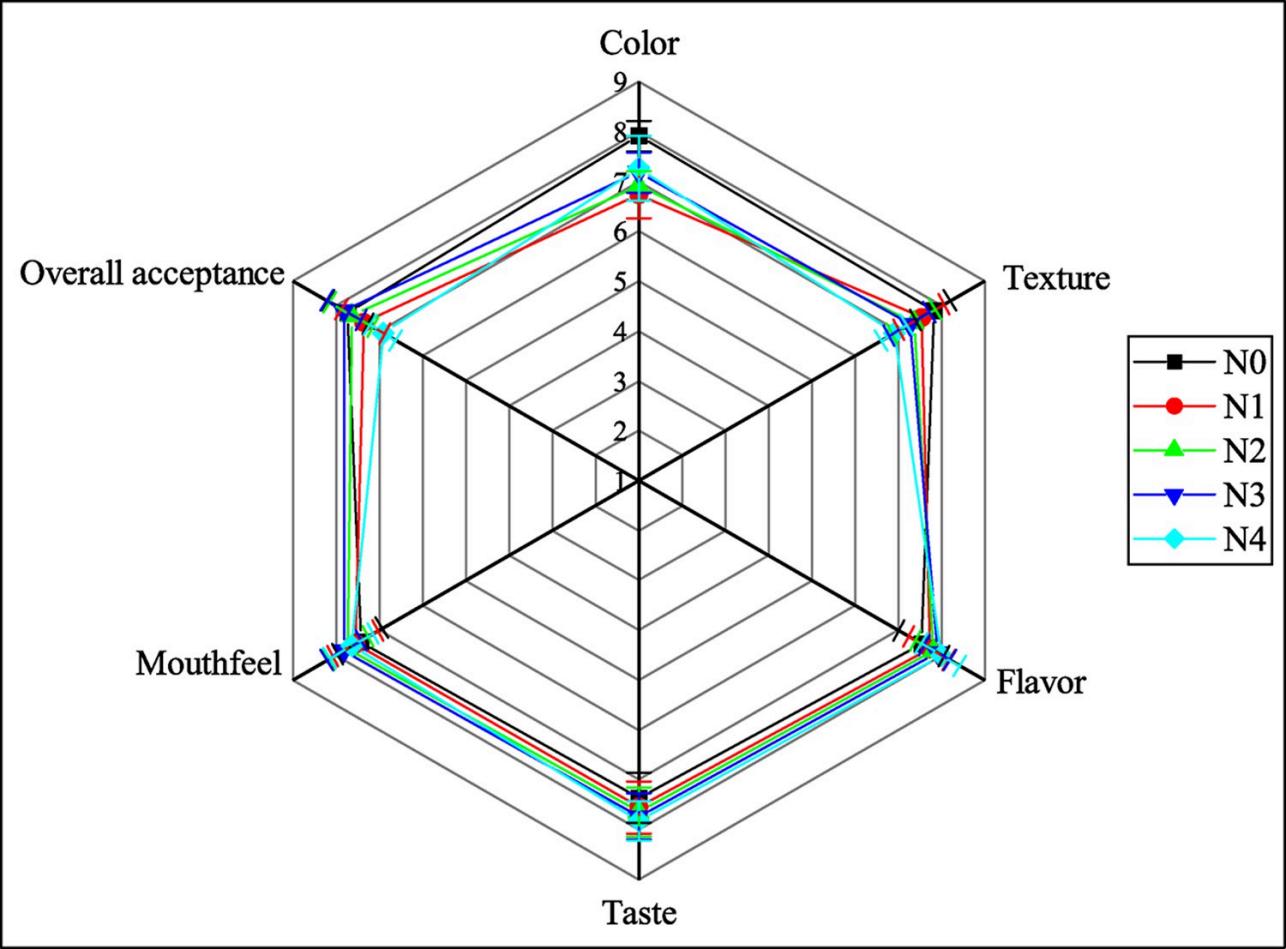
Sensory attributes of noodles. Values are expressed as mean ± SD (n  =  11). Scale: 9-point Hedonic; Here, N0 = 100% WF; N1 = 80% WF + 10% GBF + 10% FMF; N2 = 70% WF + 10% GBF + 20% FMF; N3 = 60% WF + 10% GBF + 30% FMF; N4 = 50% WF + 10% GBF + 40% FMF.

The sensory characteristics of the noodles are depicted in Fig 7. Color plays a pivotal role in capturing the consumer’s attention, impacting their perception of a product’s quality and overall impression, and aligning with human aesthetic preferences. The color of the noodles underwent changes with the substitution of FMF and GBF. Noodles made solely from wheat flour exhibited the highest color value (7.91 ± 0.30), which decreased with the introduction of 10% FMF and 10% GBF. However, as the proportion of FMF increased, the color value ascended again. Notably, N1 noodles had the lowest color value of 6.73 ± 0.47, while the N4 noodles scored 7.27 ± 0.65. The substitution of FMF at higher levels directly impacted the texture of the noodles. While the texture value of the N0 (7.82 ± 0.40) was not significantly higher compared to N1 to N3 noodles (ranging from 7.55 ± 0.52 to 7.27 ± 0.47), the N4 noodles scored significantly lower at 6.91 ± 0.30. FM is known to be a hardy crop, but the process of preparing the dough was challenging. Moreover, using more than 30% FMF made the noodle strands prone to breaking due to their hardness.

Variability in flavor and taste was observed among the noodles. The taste score of N3 noodles was 7.73 ± 0.47, not substantially different from the N0 (7.36 ± 0.50), N1 (7.55 ± 0.52), N2 (7.64 ± 0.50), and N4 noodles (7.82 ± 0.40). The addition of FMF and GBF improved the taste rating of the developed noodles from 7.73 ± 0.47 to 8.00 ± 0.45 compared to the N0 (7.55 ± 0.52). The mouthfeel of N1, N2, N3, and the N0)did not show any significant statistical differences. The overall acceptance score for N3 noodles was 7.82 ± 0.40, which was higher than that of N0 (7.73 ± 0.47), N1 (7.36 ± 0.50), N2 (7.64 ± 0.50), and N4 (6.91 ± 0.30). These findings suggest that various types of noodles can be produced by incorporating up to 30% FMF, potentially enhancing the nutritional content of the food and reducing reliance on wheat flour. Meherunnahar et al. [1] found that FTM flour impacted the noodles’ colour, flavour, texture, taste, and overall acceptability. Noodles made from wheat flour received the highest colour and texture score but declined considerably (p < 0.05) with higher FTM flour levels. FTM, 5% mushroom, and 5% rice bran flour improved the noodles’ flavour and taste. No significant difference (p < 0.05) in overall acceptability was found between FTM (30–50%) and control (100% wheat flour) noodles. Up to 50% FTM flour can be used to make noodles and baked goods.

## Conclusions

Overall, the study’s investigation into substituting 10–40% FMF and 10% GBF for wheat flour in noodle production (N1, N2, N3, and N4) demonstrates promising results. Both FMF and GBF proved effective components in noodle recipes, resulting in significant improvements in the nutritional and functional properties of the noodles. The addition of FMF and GBF notably increased the noodles’ prebiotic potential and antioxidant capacity, indicating potential health benefits. Additionally, sensory evaluations showed that noodles fortified with 30% FMF and 10% GBF had acceptability ratings comparable to conventional wheat flour noodles, suggesting good palatability. To summarize, substituting 30% FMF and 10% GBF for wheat flour in noodle production could create healthier noodles with prebiotic effects on beneficial bacteria such as *Lactobacillus acidophilus*, *Lactobacillus rhamnosus*, and *Lactobacillus plantarum*. These results highlight the potential to develop functional noodle products that offer both nutritional benefits and promote intestinal health. The future research plan will entail employing Response Surface Methodology (RSM) techniques to optimize the processing conditions, evaluating the sensory attributes and consumer acceptance to inform marketing strategies, conducting extended intervention and bioavailability studies to assess the long-term effects of consuming these fortified noodles, and determining the cost implications of incorporating foxtail millet and green banana flour.

## Supporting information

S1 Table The growth kinetics of probiotics bacteria in MRS medium supplemented with prebiotics: (A) *L*. *plantarum*, (B) *L*. *rhamnosus* and (C) *L*. *acidophilus*. (PDF)

S2 Table Nutrient and mineral composition of noodles. (PDF)

S3 Table Color intensity (L*a*b*) of noodles. (PDF)

S4 Table Cooking quality (cooking loss, water uptake and cooking yield) of noodles. (PDF)

S5 Table Water activity of noodles. (PDF)

S6 Table Antioxidant activity (DPPH radical scavenging activity and FRAP assay) of noodles extract. (PDF)

S7 Table Antioxidant characteristics (TPC and TAC) of noodles. (PDF)

S8 Table Sensory attributes of noodles. (PDF)
