# Supplementary material for: Enhancing prebiotic, antioxidant, and nutritional qualities of noodles: A collaborative strategy with foxtail millet and green banana flour
Source: PLoS One. 2024 Aug 19;19(8):e0307909. doi: 10.1371/journal.pone.0307909 (PMC11332954; doi:10.1371/journal.pone.0307909)
Supplement: S1 Table — (PDF) [file pone.0307909.s001.pdf]

Table 1 The growth kinetics of probiotics bacteria in MRS medium supplemented with prebiotics: (A) *L. plantarum*, (B) *L. rhamnosus* and (C) *L. acidophilus*

(A) *Lactobacillus plantarum*

**Sample**	**After 24 h**					**After 48 h**					**After 72 h**					**After 96 h**				
	OD			Average	STD	OD			Average	STD	OD			Average	STD	OD			Average	STD
MRS	0.55	0.56	0.55	0.55	0.01	0.55	0.56	0.55	0.55	0.01	0.55	0.56	0.55	0.55	0.01	0.55	0.56	0.55	0.55	0.01
MRS + L.p.	0.73	0.75	0.76	0.75	0.02	0.94	0.90	0.93	0.92	0.02	0.81	0.83	0.80	0.81	0.02	0.62	0.61	0.62	0.62	0.01
MRS + L.p. + GB	1.50	1.40	1.40	1.43	0.06	1.80	1.70	1.70	1.73	0.06	0.90	1.00	1.00	0.97	0.06	0.80	0.82	0.84	0.82	0.02
MRS + L.p. + FMF	1.90	1.80	1.90	1.87	0.06	2.00	2.00	2.10	2.03	0.06	1.40	1.00	1.00	1.13	0.23	0.78	0.88	0.88	0.85	0.06
MRS + L.p. + N3 without salt	1.40	1.40	1.50	1.43	0.06	1.80	1.70	1.70	1.73	0.06	0.90	1.00	1.00	0.97	0.06	0.80	0.82	0.84	0.82	0.02
MRS + L.p. + N3 with salt	1.10	1.00	1.20	1.10	0.10	1.60	1.70	1.70	1.67	0.06	1.00	1.00	0.80	0.93	0.12	0.90	0.92	0.87	0.90	0.03

Here, N0 = 100% WF; N1 = 80% WF + 10% GBF + 10% FMF; N2 = 70% WF + 10% GBF + 20% FMF; N3 = 60% WF + 10% GBF + 30% FMF; N4 = 50% WF + 10% GBF + 40% FMF and MRS = media (only); MRS + L.p. = Media + *Lactobacillus plantarum* bacteria; MRS + L.p. + GB = Media + *Lactobacillus plantarum* bacteria + 10% Green Banana Flour; MRS + L.p. + FMF = Media + *Lactobacillus plantarum* bacteria + 10% Foxtail Millet Flour; MRS + L.p. + N3 without salt = Media + *Lactobacillus plantarum* bacteria + N3 Noodles without salt; MRS + L.p. + N3 with salt = Media + *Lactobacillus plantarum* bacteria + N3 Noodles with salt

(B) *Lactobacillus ramnosus*

Sample	After 24 h					After 48 h					After 72 h					After 96 h				
	OD			Average	STD	OD			Average	STD	OD			Average	STD	OD			Average	STD
MRS	0.55	0.56	0.55	0.55	0.01	0.55	0.56	0.55	0.55	0.01	0.55	0.56	0.55	0.55	0.01	0.55	0.56	0.55	0.55	0.01
MRS + L.r.	0.61	0.61	0.60	0.61	0.01	0.82	0.81	0.80	0.81	0.01	0.69	0.68	0.69	0.69	0.01	0.53	0.51	0.50	0.51	0.02
MRS + L.r. + GB	1.40	1.20	1.20	1.27	0.12	1.70	1.50	1.70	1.63	0.12	1.00	0.90	0.90	0.93	0.06	0.78	0.75	0.76	0.76	0.02
MRS + L.r. + FMF	1.60	1.40	1.60	1.53	0.12	1.90	1.70	1.80	1.80	0.10	1.20	1.00	1.10	1.10	0.10	0.90	0.88	0.87	0.88	0.02
MRS + L.r. + N3 without salt	1.30	1.20	1.30	1.27	0.06	1.90	1.70	1.80	1.80	0.10	1.20	1.10	1.00	1.10	0.10	0.72	0.72	0.74	0.73	0.01
MRS + L.r. + N3 with salt	1.00	1.00	1.10	1.03	0.06	1.40	1.30	1.30	1.33	0.06	0.90	1.00	0.78	0.89	0.11	0.60	0.62	0.70	0.64	0.05

Here, N0 = 100% WF; N1 = 80% WF + 10% GBF + 10% FMF; N2 = 70% WF + 10% GBF + 20% FMF; N3 = 60% WF + 10% GBF + 30% FMF; N4 = 50% WF + 10% GBF + 40% FMF and MRS = media (only); MRS + L.r. = Media + *Lactobacillus ramnosus* bacteria; MRS + L.r. + GB = Media + *Lactobacillus ramnosus* bacteria + 10% Green Banana Flour; MRS + L.r. + FMF = Media + *Lactobacillus ramnosus* bacteria + 10% Foxtail Millet Flour; MRS + L.r. + N3 without salt = Media + *Lactobacillus ramnosus* bacteria + N3 Noodles without salt; MRS + L.r. + N3 with salt = Media + *Lactobacillus ramnosus* bacteria + N3 Noodles with salt

(C) *Lactobacillus acidophilus*

Sample	After 24 h					After 48 h					After 72 h					After 96 h				
	OD			Average	STD	OD			Average	STD	OD			Average	STD	OD			Average	STD
MRS	0.55	0.56	0.55	0.55	0.01	0.55	0.56	0.55	0.55	0.01	0.55	0.56	0.55	0.55	0.01	0.55	0.56	0.55	0.55	0.01
MRS + L.a.	0.70	0.71	0.69	0.70	0.01	0.92	0.90	0.92	0.91	0.01	0.79	0.77	0.79	0.78	0.01	0.63	0.61	0.60	0.61	0.02
MRS + L.a. + GB	1.10	1.10	1.20	1.13	0.06	1.50	1.70	1.50	1.57	0.12	0.88	1.00	0.90	0.93	0.06	0.80	0.82	0.83	0.82	0.02
MRS + L.a. + FMF	1.40	1.40	1.50	1.43	0.06	1.70	1.60	1.70	1.67	0.06	1.00	1.10	1.00	1.03	0.06	0.88	0.90	0.88	0.89	0.01
MRS + L.a. + N3 without salt	1.20	1.20	1.30	1.23	0.06	1.80	1.70	1.80	1.77	0.06	1.10	1.00	1.00	1.03	0.06	0.82	0.80	0.79	0.80	0.02
MRS + L.a. + N3 with salt	0.90	0.90	0.95	0.92	0.03	1.10	1.10	1.20	1.13	0.06	0.90	1.00	0.98	0.96	0.05	0.70	0.68	0.70	0.69	0.01

Here, N0 = 100% WF; N1 = 80% WF + 10% GBF + 10% FMF; N2 = 70% WF + 10% GBF + 20% FMF; N3 = 60% WF + 10% GBF + 30% FMF; N4 = 50% WF + 10% GBF + 40% FMF and MRS = media (only); MRS + L.a. = Media + *Lactobacillus acidophilus* bacteria; MRS + L.a. + GB = Media + *Lactobacillus acidophilus* bacteria + 10% Green Banana Flour; MRS + L.a. + FMF = Media + *Lactobacillus acidophilus* bacteria + 10% Foxtail Millet Flour; MRS + L.a. + N3 without salt = Media + *Lactobacillus acidophilus* bacteria + N3 Noodles without salt; MRS + L.a. + N3 with salt = Media + *Lactobacillus acidophilus* bacteria + N3 Noodles with salt
