# Supplementary material for: Enhancing prebiotic, antioxidant, and nutritional qualities of noodles: A collaborative strategy with foxtail millet and green banana flour
Source: PLoS One. 2024 Aug 19;19(8):e0307909. doi: 10.1371/journal.pone.0307909 (PMC11332954; doi:10.1371/journal.pone.0307909)
Supplement: S2 Table — (PDF) [file pone.0307909.s002.pdf]

Table 2 Nutrient and mineral composition of noodles

Nutrients	N0			N1			N2			N3			N4		
	Value	Average	STD	Value	Average	STD	Value	Average	STD	Value	Average	STD	Value	Average	STD
Moisture (g/100 g)	5.17	5.31	0.13	5.47	5.68	0.20	6.00	6.40	0.36	7.93	7.80	0.11	7.95	8.09	0.13
	5.43			5.72			6.50			7.72			8.20		
	5.34			5.86			6.70			7.76			8.12		
Ash (g/100 g)	1.47	1.54	0.06	1.94	2.00	0.09	1.99	2.10	0.11	2.19	2.20	0.06	2.25	2.29	0.05
	1.57			2.11			2.20			2.26			2.28		
	1.59			1.96			2.11			2.14			2.35		
Protein (g/100 g)	11.69	11.80	0.10	11.72	11.84	0.11	12.09	11.90	0.18	12.11	11.95	0.17	11.91	12.10	0.11
	11.89			11.93			11.75			11.97			12.01		
	11.83			11.87			11.86			11.77			12.12		
Fat (g/100 g)	1.04	1.23	0.17	1.20	1.30	0.13	1.48	1.56	0.01	1.90	1.82	0.07^{0}	1.98	2.09	0.13
	1.38			1.26			1.52			1.77			2.06		
	1.27			1.44			1.67			1.80			2.23		
Crude fiber (g/100 g)	0.15	0.18	0.03	0.52	0.49	0.03	0.83	0.71	0.11	1.01	0.92	0.08	1.10	1.14	0.18
	0.18			0.47			0.62			0.86			0.99		
	0.20			0.48			0.67			0.89			1.34		
Carbohydrate (g/100 g)	80.64	80.12	0.46	79.68	79.18	0.44	78.44	78.05	0.39	75.88	76.23	0.33	75.92	75.52	0.38
	79.74			78.99			78.03			76.28			75.45		
	79.98			78.86			77.67			76.53			75.18		
Energy (Kcal) (Kcal/100 g)	378.66	378.75	0.16	376.37	375.75	0.71	375.48	373.79	1.46	369.00	369.10	0.25	369.11	368.91	0.49
	378.93			374.97			372.82			368.91			368.36		
	378.65			375.91			373.09			369.38			369.28		
Sodium (mg/100 g)	334.60	341.82	7.90	347.1	339.89	8.81	344.11	339.72	6.64	350.81	340.73	9.30	349.03	341.12	7.14
	350.25			330.1			332.08			332.49			335.16		
	340.60			342.5			342.96			338.89			339.17		

Potassium (mg/100 g)	311.65	304.43	7.01	308.2	315.21	6.07	310.54	318.23	8.82	309.91	319.89	8.84	313.08	321.56	10.22
	303.98			319.0			316.29			323.03			332.91		
	297.65			318.4			327.85			326.74			318.69		
Calcium (mg/100 g)	152.23	148.98	9.72	149.8	152.42	8.42	151.22	153.62	8.74	156.52	155.28	7.44	150.39	156.33	7.29
	138.05			161.9			146.34			147.29			164.47		
	156.66			145.7			163.31			162.02			154.13		
Magnesium (mg/100 g)	35.56	40.89	6.73	53.3	60.21	6.45	56.65	63.57	7.99	72.79	66.35	5.70	66.08	71.01	5.81
	38.65			61.2			61.74			64.3			69.54		
	48.45			66.1			72.31			61.95			77.41		
Iron (mg/100 g)	2.42	2.33	0.10	2.9	2.97	0.11	3.47	3.59	0.17	3.69	3.73	0.17	4.53	4.34	0.18
	2.33			3.1			3.52			3.92			4.18		
	2.23			3.0			3.78			3.58			4.30		
Copper (mg/100 g)	0.65	0.67	0.05	3.3	3.52	0.22	3.69	3.88	0.26	4.34	4.04	0.28	4.53	4.37	0.18
	0.63			3.8			3.77			3.79			4.41		
	0.72			3.5			4.18			3.98			4.17		
Zinc (mg/100 g)	0.36	0.44	0.07	0.4	0.48	0.09	0.55	0.52	0.10	0.47	0.55	0.09	0.51	0.59	0.09
	0.46			0.4			0.41			0.65			0.69		
	0.49			0.6			0.61			0.54			0.56		
Manganese (mg/100 g)	0.79	0.86	0.07	2.5	2.67	0.16	2.66	2.78	0.18	2.85	3.07	0.20	3.71	3.85	0.16
	0.92			2.8			2.99			3.23			4.02		
	0.88			2.7			2.69			3.13			3.82		
Dietary Fiber (g/100 g)	7.48	7.33	0.13	9.21	9.37	0.14	10.79	10.96	0.16	11.79	11.88	0.11	12.67	12.71	0.12
	7.22			9.41			11.09			11.85			12.85		
	7.29			9.49			11.01			12.01			12.62		

Here, N0 = 100% WF; N1 = 80% WF + 10% GBF + 10% FMF; N2 = 70% WF + 10% GBF + 20% FMF; N3 = 60% WF + 10% GBF + 30% FMF; N4 = 50% WF + 10% GBF + 40% FMF
