# Supplementary material for: Enhancing prebiotic, antioxidant, and nutritional qualities of noodles: A collaborative strategy with foxtail millet and green banana flour
Source: PLoS One. 2024 Aug 19;19(8):e0307909. doi: 10.1371/journal.pone.0307909 (PMC11332954; doi:10.1371/journal.pone.0307909)
Supplement: S3 Table — (PDF) [file pone.0307909.s003.pdf]

Table 3 Color intensity (L*a*b*) of noodles

Sample	L*			a*			b*		
	Value	Average	STD	Value	Average	STD	Value	Average	STD
N0	85.73	85.63	0.30	-1.23	-1.23	0.01	10.51	10.50	0.01
	85.86			-1.23			10.49		
	85.29			-1.22			10.50		
N1	79.17	79.30	0.12	-0.15	-0.15	0.01	7.46	7.45	0.01
	79.40			-0.15			7.44		
	79.33			-0.16			7.44		
N2	79.82	79.75	0.08	-0.27	-0.26	0.01	8.21	8.20	0.01
	79.76			-0.27			8.19		
	79.66			-0.25			8.21		
N3	79.78	79.77	0.10	-0.28	-0.30	0.02	8.78	8.78	0.01
	79.86			-0.31			8.79		
	79.66			-0.30			8.78		
N4	80.83	80.94	0.11	-0.62	-0.62	0.01	8.93	8.93	0.01
	81.04			-0.61			8.94		
	80.94			-0.62			8.92		

Here, N0 = 100% WF; N1 = 80% WF + 10% GBF + 10% FMF; N2 = 70% WF + 10% GBF + 20% FMF; N3 = 60% WF + 10% GBF + 30% FMF; N4 = 50% WF + 10% GBF + 40% FMF
