# Supplementary material for: Enhancing prebiotic, antioxidant, and nutritional qualities of noodles: A collaborative strategy with foxtail millet and green banana flour
Source: PLoS One. 2024 Aug 19;19(8):e0307909. doi: 10.1371/journal.pone.0307909 (PMC11332954; doi:10.1371/journal.pone.0307909)
Supplement: S4 Table — (PDF) [file pone.0307909.s004.pdf]

Table 4 Cooking quality (cooking loss, water uptake and cooking yield) of noodles

Sample	Cooking loss (%)			Water uptake (%)			Cooking Yield (%)		
	Value	Aver.	STD	Value	Aver.	STD	Value	Aver.	STD
N0	11.53	11.14	0.45	134.89	136.23	1.73	218.94	218.88	1.66
	11.23			138.18			220.52		
	10.65			135.61			217.19		
N1	9.42	9.89	0.41	164.83	163.06	2.45	264.83	263.06	2.45
	10.07			164.10			264.10		
	10.18			160.27			260.27		
N2	9.14	9.28	0.17	168.62	166.28	2.54	268.62	266.28	2.54
	9.47			163.58			263.58		
	9.23			166.63			266.63		
N3	9.97	10.33	0.36	171.32	171.44	1.26	271.32	271.44	1.26
	10.69			170.23			270.23		
	10.34			172.75			272.75		
N4	11.96	11.76	0.65	173.57	173.97	1.62	273.57	273.97	1.62
	12.29			172.59			272.59		
	11.03			175.75			275.75		

Here, N0 = 100% WF; N1 = 80% WF + 10% GBF + 10% FMF; N2 = 70% WF + 10% GBF + 20% FMF; N3 = 60% WF + 10% GBF + 30% FMF; N4 = 50% WF + 10% GBF + 40% FMF
