# Supplementary material for: Enhancing prebiotic, antioxidant, and nutritional qualities of noodles: A collaborative strategy with foxtail millet and green banana flour
Source: PLoS One. 2024 Aug 19;19(8):e0307909. doi: 10.1371/journal.pone.0307909 (PMC11332954; doi:10.1371/journal.pone.0307909)
Supplement: S5 Table — (PDF) [file pone.0307909.s005.pdf]

Table 5 Water activity of Noodles

Sample	Water activity (a_w)		
	Value	Average	STD
N0	0.408	0.405	0.003
	0.406		
	0.402		
N1	0.375	0.378	0.003
	0.381		
	0.377		
N2	0.363	0.366	0.004
	0.365		
	0.371		
N3	0.349	0.352	0.003
	0.355		
	0.351		
N4	0.343	0.345	0.002
	0.346		
	0.347		

Here, N0 = 100% WF; N1 = 80% WF + 10% GBF + 10% FMF; N2 = 70% WF + 10% GBF + 20% FMF; N3 = 60% WF + 10% GBF + 30% FMF; N4 = 50% WF + 10% GBF + 40% FMF
