# Supplementary material for: Enhancing prebiotic, antioxidant, and nutritional qualities of noodles: A collaborative strategy with foxtail millet and green banana flour
Source: PLoS One. 2024 Aug 19;19(8):e0307909. doi: 10.1371/journal.pone.0307909 (PMC11332954; doi:10.1371/journal.pone.0307909)
Supplement: S6 Table — (PDF) [file pone.0307909.s006.pdf]

Table 6 Antioxidant activity (DPPH radical scavenging activity and FRAP assay) of noodles extract

Sample	FRAP Inhibition (%)			DPPH free radical scavenging activity (%)		
	Value	Average	STD	Value	Average	STD
N0	61.42	61.37	0.85	8.45	8.44	0.41
	62.19			8.03		
	60.49			8.84		
N1	78.89	79.42	0.66	23.19	22.10	1.16
	79.22			20.88		
	80.15			22.23		
N2	82.08	81.81	0.87	31.49	30.88	0.57
	80.83			30.79		
	82.51			30.36		
N3	84.82	84.67	0.59	37.38	37.23	0.76
	84.03			37.90		
	85.17			36.40		
N4	87.28	86.85	0.38	42.79	42.78	0.77
	86.69			42.01		
	86.59			43.55		

Here, N0 = 100% WF; N1 = 80% WF + 10% GBF + 10% FMF; N2 = 70% WF + 10% GBF + 20% FMF;

N3 = 60% WF + 10% GBF + 30% FMF; N4 = 50% WF + 10% GBF + 40% FMF
