# Supplementary material for: Enhancing prebiotic, antioxidant, and nutritional qualities of noodles: A collaborative strategy with foxtail millet and green banana flour
Source: PLoS One. 2024 Aug 19;19(8):e0307909. doi: 10.1371/journal.pone.0307909 (PMC11332954; doi:10.1371/journal.pone.0307909)
Supplement: S7 Table — (PDF) [file pone.0307909.s007.pdf]

Table 7 Antioxidant characteristics (TPC and TAC) of noodles

Sample Code	Total phenolic content (TPC) (mg/100 g)			Total antioxidant content (TAC) (mg/100 g)		
	Value	Average	STD	Value	Average	STD
N0	4.82	4.66	0.19	110.134	108.73	1.98
	4.71			106.465		
	4.44			109.586		
N1	16.45	17.81	1.19	170.355	172.57	2.08
	18.64			174.495		
	18.33			172.845		
N2	23.28	23.33	0.24	210.715	208.53	2.34
	23.58			208.811		
	23.12			206.057		
N3	27.21	28.44	1.20	239.278	241.19	2.04
	28.50			240.940		
	29.61			243.338		
N4	37.43	36.35	0.98	277.416	274.94	2.51
	36.10			272.394		
	35.52			275.022		

Here, N0 = 100% WF; N1 = 80% WF + 10% GBF + 10% FMF; N2 = 70% WF + 10% GBF + 20% FMF; N3 = 60% WF + 10% GBF + 30% FMF; N4 = 50% WF + 10% GBF + 40% FMF
