# Supplementary material for: Enhancing prebiotic, antioxidant, and nutritional qualities of noodles: A collaborative strategy with foxtail millet and green banana flour
Source: PLoS One. 2024 Aug 19;19(8):e0307909. doi: 10.1371/journal.pone.0307909 (PMC11332954; doi:10.1371/journal.pone.0307909)
Supplement: S8 Table — (PDF) [file pone.0307909.s008.pdf]

Table 8 Sensory attributes of noodles

Sample	Color												
	**Fe-1**	**Fe-2**	**Fe-3**	**Fe-4**	**Fe-5**	**Fe-6**	**M-7**	**M-8**	**M-9**	**M-10**	**M-11**	**Average**	**STD**
N0	8	8	7	8	8	8	8	8	8	8	8	7.91	0.30
N1	7	7	6	7	7	7	7	7	7	6	6	6.73	0.47
N2	6	7	7	7	7	7	7	7	7	7	7	6.91	0.30
N3	7	7	7	7	8	7	8	7	7	7	7	7.18	0.40
N4	7	6	7	7	8	7	7	8	8	7	8	7.27	0.65

Sample	Texture												
	**Fe-1**	**Fe-2**	**Fe-3**	**Fe-4**	**Fe-5**	**Fe-6**	**M-7**	**M-8**	**M-9**	**M-10**	**M-11**	**Average**	**STD**
N0	8	7	8	8	8	7	8	8	8	8	8	7.82	0.40
N1	7	7	8	7	8	8	7	8	8	7	8	7.55	0.52
N2	8	8	8	7	7	7	8	7	7	7	7	7.36	0.50
N3	7	7	7	7	7	8	7	7	7	8	8	7.27	0.47
N4	6	7	7	7	7	7	7	7	7	7	7	6.91	0.30

Sample	Flavor												
	Fe-1	Fe-2	Fe-3	Fe-4	Fe-5	Fe-6	M-7	M-8	M-9	M-10	M-11	Average	STD
N0	7	7	8	8	7	8	7	8	8	8	7	7.55	0.52
N1	8	8	8	7	8	8	8	7	8	7	8	7.73	0.47
N2	8	8	7	8	7	8	8	8	8	8	8	7.82	0.40
N3	8	8	8	7	8	8	8	8	8	8	8	7.91	0.30
N4	8	8	8	8	7	9	8	8	8	8	8	8.00	0.45

Sample	Taste												
	Fe-1	Fe-2	Fe-3	Fe-4	Fe-5	Fe-6	M-7	M-8	M-9	M-10	M-11	Average	STD
N0	7	8	7	7	7	8	7	8	7	7	8	7.36	0.50
N1	8	7	7	8	8	7	8	7	8	8	7	7.55	0.52
N2	8	8	8	7	8	7	8	7	8	7	8	7.64	0.50
N3	8	8	8	8	8	7	8	8	7	8	7	7.73	0.47
N4	8	8	8	8	8	7	8	8	8	7	8	7.82	0.40

Sample	Mouthfeel												
	**Fe-1**	**Fe-2**	**Fe-3**	**Fe-4**	**Fe-5**	**Fe-6**	**M-7**	**M-8**	**M-9**	**M-10**	**M-11**	**Average**	**STD**
N0	7	8	7	8	7	7	8	7	8	7	8	7.45	0.52
N1	8	8	7	8	7	7	7	7	8	8	8	7.55	0.52
N2	8	7	8	8	8	8	8	8	7	8	7	7.73	0.47
N3	8	8	8	8	7	8	7	8	8	8	8	7.82	0.40
N4	8	8	8	8	7	7	8	8	7	7	8	7.64	0.50

Sample	Overall acceptance												
	**Fe-1**	**Fe-2**	**Fe-3**	**Fe-4**	**Fe-5**	**Fe-6**	**M-7**	**M-8**	**M-9**	**M-10**	**M-11**	**Average**	**STD**
N0	7	8	7	7	8	8	8	8	8	8	8	7.73	0.47
N1	8	8	7	7	7	7	7	8	7	8	7	7.36	0.50
N2	7	7	8	8	8	8	7	8	8	7	8	7.64	0.50
N3	7	8	8	8	8	8	8	7	8	8	8	7.82	0.40
N4	7	7	6	7	7	7	7	7	7	7	7	6.91	0.30

Here, Fe-1 to Fe-6 = Female panel member; M-7 to M-11 = Male panel member;

N0 = 100% WF; N1 = 80% WF + 10% GBF + 10% FMF; N2 = 70% WF + 10% GBF + 20% FMF; N3 = 60% WF + 10% GBF + 30% FMF; N4 = 50% WF + 10% GBF + 40% FMF
